# Supplementary material for: Use and users of a social science research data archive
Source: PLoS One. 2020 Aug 6;15(8):e0233455. doi: 10.1371/journal.pone.0233455 (PMC7410240; doi:10.1371/journal.pone.0233455)
Supplement: Attachment — Submitted filename: Reviewers.comments.docx [file pone.0233455.s001.docx]

Reviewer’s Comment

Author’s in the manuscript entitled “Use and users of a social science research” investigates about the number and type of downloaded datasets from Finish social science research data archive. Author’s also elaborate about the number of citations for data, the demographics of data downloads and the purposes of data download. However, author’s needs to justify the following comments and the manuscript is not ready for publications yet.

1. Author need to justify the statement in line number 122, “the reuse of quantitative data is far more typical than reuse of qualitative data”.
2. Author need to justify as to why certain data download involves registration while others do not.
3. Authors have used two contradictory statements. In line number 177, it mentioned as “the number of cases (N) varies in our data between 7566-10346”. However, in line number 178, 179, it is mentioned as, “in total data contains 10346 cases”. How many total data cases are there in FSD database?
4. Table 1 is missing from the manuscript. Authors are required to include the table in the manuscript.
